# Baseline thyroid function and treatment-emergent thyroid dysfunction predict pathological response and survival after neoadjuvant PD-1 inhibitor plus platinum-based chemotherapy in locally advanced gastric and gastroesophageal junction adenocarcinoma: a multicenter cohort study

**DOI:** 10.3389/fendo.2026.1872057

**Published:** 2026-07-27

**Authors:** Zhiqiang Wang, Jie Zheng, Le Wang, Ning Meng, Zhenjiang Guo, Xiaolong Li, Kaixuan Gao, Tao Zheng

**Affiliations:** 1 Department of Gastrointestinal Surgery, Shijiazhuang People’s Hospital, Shijiazhuang, Hebei, China; 2 Department of Ultrasound, Shijiazhuang People’s Hospital, Shijiazhuang, Hebei, China; 3 Department of General Surgery, Hengshui People’s Hospital, Hengshui, Hebei, China; 4 Department of General Surgery, Baoding Central Hospital, Baoding, Hebei, China; 5 Department of General Surgery, Cangzhou People’s Hospital, Cangzhou, Hebei, China; 6 The Third Department of Surgery, The Fourth Hospital of Hebei Medical University, Shijiazhuang, Hebei, China

**Keywords:** gastric cancer, gastroesophageal junction adenocarcinoma, neoadjuvant immunotherapy, nomogram, pathological complete response, PD-1 inhibitor, thyroid function, treatment-emergent thyroid dysfunction

## Abstract

**Background:**

Thyroid dysfunction is among the most frequent endocrine immune-related adverse events (irAEs) during PD-1/PD-L1 blockade, but whether baseline thyroid function and treatment-emergent thyroid dysfunction (TeTD) predict pathological response and survival after neoadjuvant immunochemotherapy in locally advanced gastric or gastroesophageal junction adenocarcinoma (LAGC/GEJC) remains unclear.

**Methods:**

This multicenter retrospective cohort enrolled 420 patients with cT2–4bN_anyM0 LAGC/GEJC from five Hebei centers (2019–2024). All received four cycles of neoadjuvant PD-1 inhibitor plus SOX or XELOX, followed by D2 gastrectomy. Baseline TSH, FT3, FT4, TPOAb, and TgAb were measured. A favorable baseline thyroid profile was prespecified as TSH 0.55–2.50 mIU/L, FT3 ≥4.30 pmol/L, normal FT4, and negative autoantibodies. TeTD was defined as new-onset hypothyroidism, thyrotoxicosis, or biphasic thyroiditis during therapy. Logistic and Cox regression, subgroup and sensitivity analyses, and a pCR nomogram were applied.

**Results:**

Of 420 patients (median age 60, 66.9% male), 114 (27.1%) had a favorable thyroid profile and 105 (25.0%) developed TeTD; tumor-intrinsic factors were balanced. Overall pCR was 23.3% and MPR 42.6%. Favorable profile predicted higher pCR (43.0% vs. 16.0%; adjusted OR 6.08, P<0.001) and MPR (61.4% vs. 35.6%; OR 3.42, P<0.001). TeTD independently predicted higher pCR (38.1% vs. 18.4%; OR 3.26, P<0.001) and MPR. Non-thyroid irAEs and discontinuation were similar (P = 0.082). At 39.2 months median follow-up, 3-year DFS was 77.7% vs. 49.8% (TeTD vs. non-TeTD; adjusted HR 0.35, P<0.001) and 3-year OS 99.0% vs. 78.4% (HR0.11, P<0.001). A thyroid-inclusive pCR nomogram achieved AUC 0.82 (95% CI 0.77–0.86) versus 0.73 for a staging-only model (P<0.001), with adequate calibration (Hosmer–Lemeshow P = 0.84). Effects were robust across all subgroups and sensitivity analyses.

**Conclusions:**

Pretreatment thyroid function and on-treatment thyroid dysfunction are independent and complementary host-factor predictors of pathological response and long-term survival after neoadjuvant PD-1 inhibitor plus platinum-based chemotherapy in LAGC/GEJC. Incorporating these inexpensive thyroid-axis variables into pretreatment risk stratification provides additional prognostic information beyond conventional staging and tissue biomarkers.

## Introduction

Gastric cancer remains the fifth most common malignancy and the fourth leading cause of cancer-related death worldwide, accounting for approximately 1.09 million new cases and 769,000 deaths in 2020 (1). In China, more than 60% of patients present with l]ocally advanced disease (LAGC, defined as clinical stage cT2–4bN_anyM0). Gastroesophageal junction adenocarcinoma (GEJC), which shares biological and anatomical continuity with proximal gastric cancer, is increasingly managed within the same multimodality framework (2). Perioperative platinum-based chemotherapy has improved resection rates and overall survival over the past two decades (3, 4), and the addition of PD-1/PD-L1 inhibitors has further increased pathological complete response (pCR) rates to 18–24% in contemporary phase III trials, compared with 5–10% achievable with chemotherapy alone (5–12).

Despite these gains, individual benefit from neoadjuvant immunochemotherapy remains heterogeneous. Established tissue biomarkers including PD-L1 combined positive score (CPS), tumor mutational burden (TMB), and microsatellite-instability-high (MSI-H) status identify only a fraction of patients most likely to benefit, and are limited by tissue accessibility, inter-laboratory variability, and the failure to capture systemic host factors (13–15). Recent multicenter and AI-assisted efforts in Chinese cohorts have begun to integrate radiomic and biochemical signals to refine pretreatment stratification, but no inexpensive, readily available systemic biomarker has yet entered routine clinical practice (16, 17).

The thyroid axis is directly relevant to immune checkpoint blockade for two reasons. First, immune-related thyroid dysfunction is among the most common endocrine toxicities of PD-1/PD-L1 inhibitors, occurring in 10–25% of treated patients across pan-cancer cohorts, and has been reported as a positive prognostic signal in advanced-stage settings (18–22). Second, thyroid hormones modulate systemic energy metabolism, lymphocyte activation, cytokine signaling, and the inflammatory milieu that shapes antitumor immunity (23). Baseline thyroid dysfunction, low-FT3 syndrome, thyroid autoantibody positivity, and treatment-emergent thyroiditis may therefore function not only as safety signals but also as indicators of host immune responsiveness. However, most published evidence linking immune-related thyroid dysfunction with improved outcomes comes from advanced or palliative pan-cancer cohorts, in which response cannot be pathologically verified and treatment decisions are less consequential. We frame both variables as host-factor predictors rather than as agents that modify chemosensitivity: a favorable baseline profile is proposed to index preserved endocrine, metabolic, and immune competence before treatment, whereas TeTD is proposed to reflect on-treatment pharmacodynamic engagement of PD-1 blockade. The present study therefore evaluates their predictive association with pathological response and survival, and does not test whether altering thyroid hormone levels changes that response (24–28).

In the neoadjuvant gastric cancer setting, pathological response can be directly assessed on the resection specimen, making this clinical scenario particularly suitable for evaluating whether thyroid-axis biomarkers provide early predictive information. To our knowledge, no large multicenter study has interrogated baseline thyroid function and treatment-emergent thyroid dysfunction (TeTD) jointly in patients receiving SOX or XELOX combined with PD-1 inhibitor for LAGC or GEJC. We designed this multicenter retrospective cohort study with five prespecified objectives: (1) to evaluate the association between a prespecified favorable baseline thyroid profile and pathological response (pCR, MPR); (2) to evaluate the association between TeTD during neoadjuvant therapy and pathological response; (3) to characterize the relationship between TeTD and the spectrum of immune-related adverse events; (4) to assess favorable baseline thyroid profile and TeTD as independent prognostic factors for disease-free survival (DFS) and overall survival (OS); and (5) to construct and internally validate a thyroid-inclusive nomogram for individualized pCR prediction.

## Materials and methods

### Study design and patient selection

This was a multicenter retrospective cohort study conducted at five tertiary centers in Hebei Province, China: The Fourth Hospital of Hebei Medical University, Shijiazhuang People’s Hospital, Cangzhou People’s Hospital, Baoding Central Hospital, and Hengshui People’s Hospital. The protocol was reviewed and approved by the Institutional Review Board of each participating center in accordance with the Declaration of Helsinki. Written informed consent was waived because of the retrospective design; all patient data were anonymized prior to analysis.

Eligible patients met all of the following criteria: histologically confirmed gastric or gastroesophageal junction adenocarcinoma; clinical stage cT2–4bN_anyM0 according to the 8th edition AJCC/UICC TNM system, established by enhanced computed tomography (CT), endoscopic ultrasonography, and/or 18F-FDG PET-CT; receipt of four cycles of neoadjuvant therapy combining a PD-1 inhibitor with one of two platinum-based doublet regimens (SOX: oxaliplatin + S-1; XELOX: oxaliplatin + capecitabine); subsequent radical gastrectomy with D2 lymphadenectomy and available postoperative histopathology; and complete baseline serum thyroid function test results (TSH, FT3, FT4, TPOAb, TgAb) obtained within 14 days prior to neoadjuvant therapy initiation.

**Figure 1:**
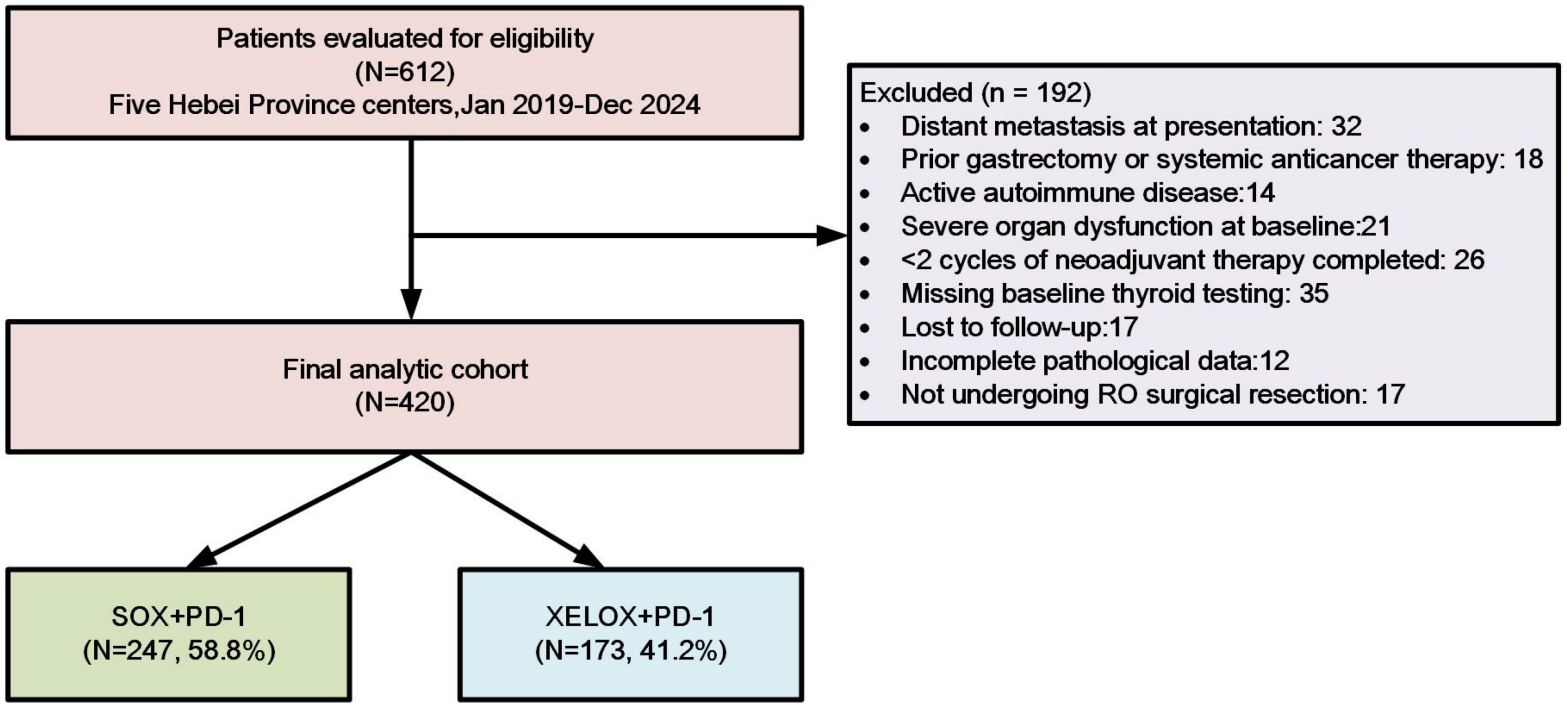
CONSORT-style patient flow diagram of the study cohort. Of 612 patients with locally advanced gastric or gastroesophageal junction adenocarcinoma evaluated at the five participating centers between January 2019 and December 2024, 192 were excluded based on prespecified criteria, leaving 420 patients in the final analytic cohort. Distribution by center: Shijiazhuang People’s Hospital (n=98), The Fourth Hospital of Hebei Medical University (n=94), Baoding Central Hospital (n=83), Hengshui People’s Hospital (n=74), and Cangzhou People’s Hospital (n=71). Distribution by neoadjuvant regimen: SOX+PD-1 (n=247, 58.8%) and XELOX+PD-1 (n=173, 41.2%). Distribution by thyroid stratifiers: favorable baseline thyroid profile (n=114, 27.1%), and treatment-emergent thyroid dysfunction (n=105, 25.0%).

Exclusion criteria were: distant metastasis at any stage; prior gastrectomy or systemic anticancer therapy; active autoimmune disease requiring ongoing immunosuppression; severe organ dysfunction at baseline; missing key clinical, pathological, or follow-up data; incomplete neoadjuvant therapy course (fewer than two cycles); and not undergoing R0 surgical resection. Of 612 patients evaluated across the five centers between January 2019 and December 2024, 420 met all inclusion criteria and constituted the final analytic cohort. Full screening and exclusion details are presented in **Figure 1**.

### Thyroid function assessment and definitions

Baseline thyroid function included serum TSH, FT3, FT4, TPOAb, and TgAb, all measured at the central laboratory of each participating hospital using locally validated chemiluminescence immunoassays. Reference ranges were harmonized across centers through the regional clinical laboratory accreditation network. A favorable baseline thyroid profile was prespecified as TSH 0.55–2.50 mIU/L, FT3 ≥4.30 pmol/L, FT4 within reference range, and negative TPOAb and TgAb. These thresholds define a prespecified favorable band rather than the boundaries of normality. The upper TSH limit of 2.50 mIU/L was selected because it marks the lower and more homogeneous portion of the euthyroid interval, within which subclinical autoimmunity and early hypothyroid drift are least frequent; TSH values of 2.50 to 4.50 mIU/L, although within the laboratory reference range, were classified as non-favorable rather than abnormal. The FT3 criterion applied a lower bound of 4.30 pmol/L with no upper cutoff, because the hypothesis concerned preserved rather than elevated FT3, and the few patients with above-range FT3 were retained in the non-favorable group. Baseline thyroid status was categorized as euthyroid, low-FT3 syndrome (FT3 <4.00 pmol/L with TSH within reference range), subclinical hypothyroidism (TSH >4.50 mIU/L with normal FT4), or subclinical hyperthyroidism (TSH <0.40 mIU/L). Thyroid function was reassessed after two cycles of neoadjuvant therapy and within 7 days before surgery. This reassessment after two cycles and again within 7 days before surgery was mandated by protocol for every patient rather than obtained selectively, so that ascertainment of treatment-emergent thyroid dysfunction was complete and uniform across the cohort.

Treatment-emergent thyroid dysfunction (TeTD) was defined as newly developed subclinical hypothyroidism, overt hypothyroidism, transient thyrotoxicosis, subclinical thyrotoxicosis, or biphasic thyroiditis arising at any point during the four cycles of neoadjuvant therapy or before surgery. Subclinical hypothyroidism required TSH >4.50 mIU/L with FT4 within reference range; overt hypothyroidism required TSH >4.50 mIU/L with FT4 below reference range. Thyrotoxicosis required TSH <0.40 mIU/L; biphasic thyroiditis was defined as a thyrotoxic phase followed by hypothyroidism within the same observation window. TeTD severity was graded according to CTCAE v5.0.

### Treatment and follow-up

All patients received four 21-day cycles of neoadjuvant therapy. SOX consisted of oxaliplatin 130 mg/m² on day 1 plus S-1 40–60 mg twice daily on days 1–14, and XELOX consisted of oxaliplatin 130 mg/m² on day 1 plus capecitabine 1000 mg/m² twice daily on days 1–14. Patients received one of five PD-1 inhibitors (camrelizumab, sintilimab, tislelizumab, pembrolizumab, or toripalimab) at standard doses according to institutional protocols and the Chinese Society of Clinical Oncology (CSCO) gastric cancer guidelines.² Radiological response was assessed by RECIST v1.1 every two cycles. D2 gastrectomy was performed 4–6 weeks after the last neoadjuvant cycle. Pathological response was assessed centrally on the resection specimen by two independent board-certified gastrointestinal pathologists at each center using the Mandard tumor regression grading (TRG) system; disagreements were resolved by a third reviewer (29). pCR was defined as Mandard TRG 1 (no residual viable tumor cells) and MPR as Mandard TRG 1–2 (≤10% residual viable tumor cells). Postoperative adjuvant therapy was administered per multidisciplinary tumor-board recommendation. Patients were followed every 3 months for the first 2 years and every 6 months thereafter.

### Outcomes and definitions

The primary endpoints were pCR and DFS. Secondary endpoints included MPR, ypT0–2 status, ypN0 status, OS, and ORR and DCR assessed by RECIST version 1.1 (30). Immune-related adverse events (irAEs) were graded according to CTCAE v5.0. DFS was defined as time from surgery to disease recurrence, distant metastasis, or death from any cause; OS was defined as time from surgery to death from any cause. Patients without events were censored at the last follow-up date. Systemic immune-inflammation index (SII) and prognostic nutritional index (PNI) were calculated as previously described, and the SII–PNI score combined the two markers (0 = neither adverse, 1 = one adverse, 2 = both adverse using SII ≥600 and PNI <47 thresholds) (31, 32).

### Statistical analysis

Continuous variables were summarized as median (interquartile range, IQR) and compared using the Mann–Whitney U test. Categorical variables were summarized as frequency (%) and compared using the χ² or Fisher’s exact test. Logistic regression was used for binary outcomes (pCR, MPR, ORR, DCR, ypT0–2, ypN0). Variables with P<0.10 in univariable analysis or with established clinical relevance were entered into multivariable models. Cox proportional hazards regression was used for DFS and OS, with proportional hazards assumptions verified by Schoenfeld residuals. Subgroup interactions were tested by inclusion of multiplicative interaction terms in the Cox model. Prespecified sensitivity analyses excluded patients with subclinical hypothyroidism at baseline, low-FT3 syndrome at baseline, autoantibody-positive patients, MSI-H patients, patients with grade 3–4 irAEs, and were also performed within each chemotherapy backbone (SOX-only, XELOX-only). Because treatment-emergent thyroid dysfunction is ascertained on treatment, immortal-time bias was addressed by prespecified sensitivity analyses: a landmark analysis re-setting the survival origin at 3 and 6 months after surgery among patients event-free to each landmark, and inspection of the proportional-hazards assumption for TeTD using scaled Schoenfeld residuals, with results reported in **Supplementary Table 2**. A thyroid-inclusive nomogram for individualized pCR prediction was developed from the multivariable logistic model. Discrimination was assessed by the area under the ROC curve (AUC) and compared between models using a bootstrap-based DeLong-equivalent test (1000 replicates). Calibration was evaluated by calibration plots and the Hosmer–Lemeshow goodness-of-fit test. All P values were two-sided, with statistical significance set at P<0.05. Analyses were performed in Python 3.11 (statsmodels, lifelines, scikit-learn) and R version 4.3.3 (survival, rms, pROC, forestplot, ggplot2 packages).

## Results

### Patient characteristics

Between January 2019 and December 2024, 420 eligible patients were enrolled across the five participating centers (**Figure 1**). The cohort included 281 men (66.9%) with a median age of 60 years (IQR 54–67). Distribution by neoadjuvant regimen was SOX+PD-1 in 247 patients (58.8%) and XELOX+PD-1 in 173 patients (41.2%). Five PD-1 inhibitors were used: sintilimab (n=151), camrelizumab (n=109), tislelizumab (n=94), toripalimab (n=47), and pembrolizumab (n=19). At baseline, 114 patients (27.1%) met all criteria for a favorable thyroid profile. This favorable-profile prevalence should not be read as the euthyroid rate. At baseline, 314 patients (74.8%) were euthyroid, 80 (19.0%) had low-FT3 syndrome, and 26 (6.2%) had subclinical hypothyroidism; the favorable profile identifies the narrower optimal band within the euthyroid majority. Baseline thyroid replacement and anti-thyroid medication use was not recorded as a discrete field in the study database, so a comparison of euthyroid patients who were euthyroid *de novo* versus euthyroid on medication cannot be reconstructed from the locked dataset; this is acknowledged as a limitation. The levothyroxine and anti-thyroid drug figures reported in **Table 1** are treatment-emergent and do not reflect baseline medication use. During the four cycles of neoadjuvant therapy, 105 patients (25.0%) developed TeTD.

**Table 1:** Immune-related adverse events and TeTD characteristics.

Parameter	No TeTD (n=315)	TeTD (n=105)	P value
Any irAE	127 (40.3)	105 (100.0)	<0.001
Grade 3–4 irAE	12 (3.8)	17 (16.2)	<0.001
Treatment discontinuation due to irAE	6 (1.9)	6 (5.7)	0.082
Skin irAE	32 (10.2)	15 (14.3)	0.283
Hepatic irAE	42 (13.3)	20 (19.0)	0.156
Endocrine irAE (any)	7 (2.2)	105 (100.0)	<0.001
Pulmonary irAE	24 (7.6)	5 (4.8)	0.381
Gastrointestinal irAE	20 (6.3)	13 (12.4)	0.059
Renal irAE	15 (4.8)	3 (2.9)	0.580
TeTD subtype: Subclinical hypothyroidism	—	27 (25.7)	—
TeTD subtype: Overt hypothyroidism	—	25 (23.8)	—
TeTD subtype: Transient thyrotoxicosis	—	24 (22.9)	—
TeTD subtype: Biphasic thyroiditis	—	18 (17.1)	—
TeTD subtype: Subclinical thyrotoxicosis	—	11 (10.5)	—
Median TeTD onset cycle (IQR)	—	2 (2–3)	—
Levothyroxine required	0 (0.0)	33 (31.4)	<0.001
Anti-thyroid drug required	0 (0.0)	4 (3.8)	0.004

By definition the endocrine irAE rate is 100% in the TeTD group; non-endocrine irAEs (skin, hepatic, pulmonary, gastrointestinal, renal) were comparable between groups, indicating that TeTD does not herald global hyper-immune activation. TeTD subtypes are shown only among the 105 patients with TeTD.

Baseline characteristics by favorable thyroid profile group are summarized in **Table 2**. Tumor-intrinsic factors that drive ICI response, including cT stage (cT4a/b: 56.1% vs. 54.9%, P = 0.826), cN stage (cN2-3: 55.3% vs. 58.8%, P = 0.579), tumor size, tumor location (Upper/GEJ: 32.5% vs. 29.7%, P = 0.634), differentiation, PD-L1 CPS ≥5 (37.7% vs. 43.1%, P = 0.373), MSI-H status (7.9% vs. 7.8%, P = 1.000), and chemotherapy regimen (SOX+PD-1 57.9% vs. 59.2%, P = 0.904), were comparable between groups, supporting the validity of subsequent comparisons. Baseline characteristics by TeTD status are summarized in **Table 3**; tumor-intrinsic factors and most clinical variables were balanced, while baseline FT3 (median 4.71 vs. 4.33 pmol/L, P<0.001), TPOAb positivity (42.9% vs. 6.0%, P<0.001), and TgAb positivity (28.6% vs. 10.2%, P<0.001) were higher in patients who subsequently developed TeTD, in keeping with the known role of preexisting thyroid autoimmunity as a risk factor for ICI-induced thyroid dysfunction.

**Table 2:** Baseline characteristics by favorable thyroid profile group.

Variable	Favorable thyroid profile (n=114)	Unfavorable profile (n=306)	P value
Age (years), median (IQR)	59 (53–66)	62 (55–67)	0.08
Age ≥65 years, n (%)	35 (30.7)	112 (36.6)	0.301
Male sex, n (%)	70 (61.4)	211 (69.0)	0.162
BMI (kg/m²), median (IQR)	22.74 (21.18–25.28)	23.55 (21.30–25.52)	0.451
BMI ≥24 kg/m², n (%)	45 (39.5)	134 (43.8)	0.44
Tumor location, n (%)	nan	nan	0.042
Lower	42 (36.8)	119 (38.9)	nan
Middle	35 (30.7)	96 (31.4)	nan
Upper	32 (28.1)	56 (18.3)	nan
GEJ	5 (4.4)	35 (11.4)	nan
Upper/GEJ location, n (%)	37 (32.5)	91 (29.7)	0.634
Poor differentiation/SRC, n (%)	74 (64.9)	176 (57.5)	0.181
Borrmann III/IV, n (%)	83 (72.8)	188 (61.4)	0.039
Tumor size (cm), median (IQR)	4.31 (3.03–5.71)	4.27 (3.09–5.96)	0.695
Tumor size >5 cm, n (%)	42 (36.8)	111 (36.3)	0.91
cT4a/b, n (%)	64 (56.1)	168 (54.9)	0.826
cN2-3, n (%)	63 (55.3)	180 (58.8)	0.579
H. pylori positive, n (%)	55 (48.2)	142 (46.4)	0.743
PD-L1 CPS ≥5, n (%)	43 (37.7)	132 (43.1)	0.373
MSI-H, n (%)	9 (7.9)	24 (7.8)	1.0
Albumin (g/L), median (IQR)	40.35 (37.50–42.35)	39.90 (37.62–42.00)	0.575
SII, median (IQR)	466 (301–694)	480 (310–700)	0.77
PNI, median (IQR)	49.40 (45.55–52.75)	48.45 (45.62–51.15)	0.116
SII–PNI score 2, n (%)	18 (15.8)	54 (17.6)	0.771
Neoadjuvant regimen, n (%)	nan	nan	0.904
SOX+PD-1	66 (57.9)	181 (59.2)	nan
XELOX+PD-1	48 (42.1)	125 (40.8)	nan

BMI, body mass index; SRC, signet ring cell; CPS, combined positive score; MSI-H, microsatellite-instability high; SII, systemic immune-inflammation index; PNI, prognostic nutritional index; SOX, S-1 + oxaliplatin; XELOX, capecitabine + oxaliplatin. Continuous variables compared by Mann-Whitney U test; categorical by Fisher exact/chi-square test.

**Table 3:** Baseline characteristics by treatment-emergent thyroid dysfunction (TeTD) status.

Variable	TeTD (n=105)	No TeTD (n=315)	P value
Age (years), median (IQR)	61 (55–68)	60 (54–66)	0.513
Age ≥65 years, n (%)	37 (35.2)	110 (34.9)	1.000
Male sex, n (%)	66 (62.9)	215 (68.3)	0.339
BMI (kg/m²), median (IQR)	23.25 (21.03–25.39)	23.53 (21.36–25.32)	0.329
BMI ≥24 kg/m², n (%)	42 (40.0)	137 (43.5)	0.570
Upper/GEJ location, n (%)	27 (25.7)	101 (32.1)	0.271
Poor differentiation/SRC, n (%)	64 (61.0)	186 (59.0)	0.819
Tumor size (cm), median (IQR)	4.47 (3.26–6.15)	4.22 (3.05–5.70)	0.306
cT4a/b, n (%)	55 (52.4)	177 (56.2)	0.499
cN2-3, n (%)	51 (48.6)	192 (61.0)	0.030
PD-L1 CPS ≥5, n (%)	51 (48.6)	124 (39.4)	0.110
MSI-H, n (%)	6 (5.7)	27 (8.6)	0.408
Baseline TSH (mIU/L), median (IQR)	2.18 (1.61–2.87)	2.38 (1.80–3.17)	0.038
Baseline FT3 (pmol/L), median (IQR)	4.71 (4.40–5.04)	4.33 (3.99–4.70)	<0.001
Baseline FT4 (pmol/L), median (IQR)	15.64 (14.57–16.51)	15.19 (14.15–16.07)	0.014
TPOAb positive, n (%)	45 (42.9)	19 (6.0)	<0.001
TgAb positive, n (%)	30 (28.6)	32 (10.2)	<0.001
Favorable thyroid profile, n (%)	28 (26.7)	86 (27.3)	1.000
SII, median (IQR)	408 (276–636)	482 (319–706)	0.135
PNI, median (IQR)	49.50 (46.60–52.00)	48.40 (45.55–51.40)	0.078
XELOX+PD-1 regimen, n (%)	42 (40.0)	131 (41.6)	0.819

TeTD, treatment-emergent thyroid dysfunction; TPOAb, thyroid peroxidase antibody; TgAb, thyroglobulin antibody. The right-shift in baseline FT3, FT4 and the marked elevation in autoantibody positivity in patients who later developed TeTD is consistent with subclinical thyroid autoimmunity priming the response.

### Pathological efficacy

The overall pCR rate was 23.3% (98/420) and the MPR rate was 42.6% (179/420), aligned with contemporary phase III data on neoadjuvant ICI plus chemotherapy in LAGC/GEJC (5, 6). As shown in **Table 4**, patients with a favorable baseline thyroid profile achieved superior pathological responses across every endpoint: pCR was 43.0% vs. 16.0% (crude P<0.001; adjusted OR 5.57, 95% CI 3.17–9.76; P<0.001) (this estimate is adjusted for clinicopathologic and tissue-biomarker covariates but not for TeTD; the fully mutually adjusted model that additionally includes TeTD, shown in **Table 5**, yields an adjusted OR of 6.08, 95% CI 3.39–10.92, which is the value quoted in the Abstract), MPR was 61.4% vs. 35.6% (adjusted OR 3.42, 95% CI 2.11–5.52; P<0.001), ORR was 64.9% vs. 45.4% (adjusted OR 2.48; P<0.001), DCR was 97.4% vs. 91.2% (P = 0.032), ypT0–2 was 64.9% vs. 33.3% (adjusted OR 8.91; P<0.001), and ypN0 was 56.1% vs. 26.1% (adjusted OR 9.31; P<0.001). The full Mandard TRG distribution differed significantly between favorable and unfavorable groups (χ² P<0.001), with favorable-profile patients shifted toward TRG 1 (43.0% vs. 16.0%) and away from TRG 4–5 (19.3% vs. 36.2%).

**Table 4:** Pathological efficacy by favorable thyroid profile and by TeTD status.

a. By favorable baseline thyroid profile					
Outcome	Favorable (n=114)	Unfavorable (n=306)	Crude P	Adjusted OR (95% CI)	Adjusted P
pCR (TRG 1)	49 (43.0)	49 (16.0)	<0.001	5.57 (3.17–9.76)	<0.001
MPR (TRG 1–2)	70 (61.4)	109 (35.6)	<0.001	3.42 (2.11–5.52)	<0.001
ORR (CR + PR)	74 (64.9)	139 (45.4)	<0.001	2.48 (1.54–3.97)	<0.001
DCR (CR + PR + SD)	111 (97.4)	279 (91.2)	0.032	3.69 (1.09–12.55)	0.036
ypT0–2	74 (64.9)	102 (33.3)	<0.001	8.91 (4.78–16.59)	<0.001
ypN0	64 (56.1)	80 (26.1)	<0.001	9.31 (4.80–18.07)	<0.001
b. By TeTD status.					
Outcome	TeTD (n=105)	No TeTD (n=315)	Crude P	Adjusted OR (95% CI)	Adjusted P
pCR (TRG 1)	40 (38.1)	58 (18.4)	<0.001	3.24 (1.81–5.79)	<0.001
MPR (TRG 1–2)	62 (59.0)	117 (37.1)	<0.001	2.64 (1.60–4.35)	<0.001
ORR (CR + PR)	68 (64.8)	145 (46.0)	0.001	2.17 (1.33–3.54)	0.002
DCR (CR + PR + SD)	101 (96.2)	289 (91.7)	0.187	2.12 (0.71–6.39)	0.180
ypT0–2	67 (63.8)	109 (34.6)	<0.001	6.93 (3.61–13.29)	<0.001
ypN0	56 (53.3)	88 (27.9)	<0.001	4.22 (2.15–8.26)	<0.001

Adjusted ORs for favorable profile additionally adjusted for: age, sex, cT, cN, PD-L1 CPS, MSI, SII-PNI score, regimen. Adjusted ORs for TeTD additionally adjusted for: age, sex, cT, cN, PD-L1 CPS, MSI, SII-PNI score, regimen, favorable thyroid profile. pCR, pathological complete response (TRG 1); MPR, major pathological response (TRG 1-2).

**Table 5:** Univariable and multivariable logistic regression for pCR (mutually adjusted).

Variable	Univariable OR (95% CI)	Univariable P	Multivariable OR (95% CI)	Multivariable P
Favorable thyroid profile (Yes vs. No)	3.95 (2.45–6.39)	<0.001	6.08 (3.39–10.92)	<0.001
Treatment-emergent thyroid dysfunction (Yes vs. No)	2.73 (1.68–4.43)	<0.001	3.26 (1.82–5.83)	<0.001
Age (≥65 vs. <65)	0.82 (0.51–1.33)	0.425	0.64 (0.36–1.13)	0.125
Sex (Male vs. Female)	1.09 (0.67–1.77)	0.725	1.28 (0.71–2.30)	0.412
BMI (≥24 vs. <24 kg/m²)	1.19 (0.76–1.88)	0.451	—	—
cT stage (cT4a/b vs. cT2/3)	0.65 (0.41–1.02)	0.060	0.67 (0.39–1.14)	0.137
cN stage (cN2–3 vs. cN0-1)	0.63 (0.40–0.99)	0.043	0.67 (0.39–1.15)	0.151
PD-L1 (CPS ≥5 vs. <5)	3.37 (2.10–5.41)	<0.001	5.23 (2.94–9.31)	<0.001
MSI-H vs. MSS	3.50 (1.70–7.23)	<0.001	7.85 (3.11–19.78)	<0.001
SII–PNI score 2 vs. 0–1	0.84 (0.45–1.56)	0.582	0.94 (0.45–1.97)	0.880
Differentiation (Poor/SRC vs. Well/Mod)	1.46 (0.91–2.34)	0.118	1.31 (0.75–2.27)	0.344
Regimen (XELOX vs. SOX)	1.29 (0.82–2.03)	0.278	1.40 (0.81–2.41)	0.224
Tumor location (Upper/GEJ vs. Mid/Lower)	1.07 (0.66–1.75)	0.776	—	—

Multivariable model included all listed predictors entered simultaneously. OR, odds ratio; CI, confidence interval; SRC, signet ring cell.

Patients who developed TeTD likewise achieved superior pathological responses (**Table 4**): pCR 38.1% vs. 18.4% (crude P<0.001; adjusted OR 3.24, 95% CI 1.81–5.79; P<0.001), MPR 59.0% vs. 37.1% (adjusted OR 2.64; P<0.001), ORR 64.8% vs. 46.0% (adjusted OR 2.17; P = 0.002), ypT0–2 63.8% vs. 34.6% (adjusted OR 6.93; P<0.001), and ypN0 53.3% vs. 27.9% (adjusted OR 4.22; P<0.001). The TRG distribution shifted toward favorable categories in TeTD patients (χ² P<0.001), with TRG 1 in 38.1% vs. 18.4% and TRG 4–5 in 19.0% vs. 35.9%.

In multivariable logistic regression for pCR with all predictors entered simultaneously (**Table 5**), favorable baseline thyroid profile (adjusted OR 6.08, 95% CI 3.39–10.92; P<0.001) and TeTD (adjusted OR 3.26, 95% CI 1.82–5.83; P<0.001) remained the two strongest independent predictors after adjustment for cT stage, cN stage, PD-L1 CPS, MSI status, SII–PNI score, regimen, differentiation, age, and sex. Other independent predictors of pCR were MSI-H (adjusted OR 7.85; P<0.001) and PD-L1 CPS ≥5 (adjusted OR 5.23; P<0.001). The fact that favorable thyroid profile and TeTD remained independently significant after mutual adjustment indicates that these two thyroid-axis variables capture complementary, non-overlapping information about pretreatment immune-endocrine status and on-treatment immune activation.

### Immune-related adverse events

Complete irAE data were available for all 420 patients (**Table 1**). Any-grade irAEs occurred in 232 patients (55.2%) overall. By definition, all 105 TeTD patients had an endocrine irAE, while 40.3% of non-TeTD patients had at least one irAE driven by other organ systems. Importantly, when restricting analysis to non-thyroid irAEs, organ-specific events were comparable between groups: skin (14.3% vs. 10.2%, P = 0.283), hepatic (19.0% vs. 13.3%, P = 0.156), pulmonary (4.8% vs. 7.6%, P = 0.381), gastrointestinal (12.4% vs. 6.3%, P = 0.059), and renal (2.9% vs. 4.8%, P = 0.580) irAEs did not differ significantly between TeTD and non-TeTD patients. Grade 3–4 irAEs were modestly more frequent in the TeTD group (16.2% vs. 3.8%, P<0.001), reflecting the inclusion of overt hypothyroidism within the grade 3 spectrum, but treatment-discontinuation rates due to irAE were not significantly different (5.7% vs. 1.9%, P = 0.082). The most frequent TeTD subtype was subclinical hypothyroidism (n=27, 25.7% of TeTD), followed by overt hypothyroidism (n=25, 23.8%), transient thyrotoxicosis (n=24, 22.9%), biphasic thyroiditis (n=18, 17.1%), and subclinical thyrotoxicosis (n=11, 10.5%). The median onset cycle of TeTD was cycle 2 (IQR cycle 2–3), and 33 of 105 TeTD patients (31.4%) required levothyroxine replacement during the perioperative window. Within the TeTD group, pathological response did not differ materially between patients who did and did not require levothyroxine (pCR 39.4% vs. 37.5%), and levothyroxine requirement was not associated with disease-free survival (HR 1.16, 95% CI 0.49–2.73; primary-cohort analysis), indicating that standard replacement of the dysfunction did not attenuate its favorable prognostic association.

These observations indicate that TeTD predominantly reflects an organ-specific endocrine immune response rather than a global hyper-immune activation, distinguishing it from biomarkers associated with severe systemic toxicity and supporting its practical utility as a positive prognostic signal.

### Survival outcomes

**Figure 2:**
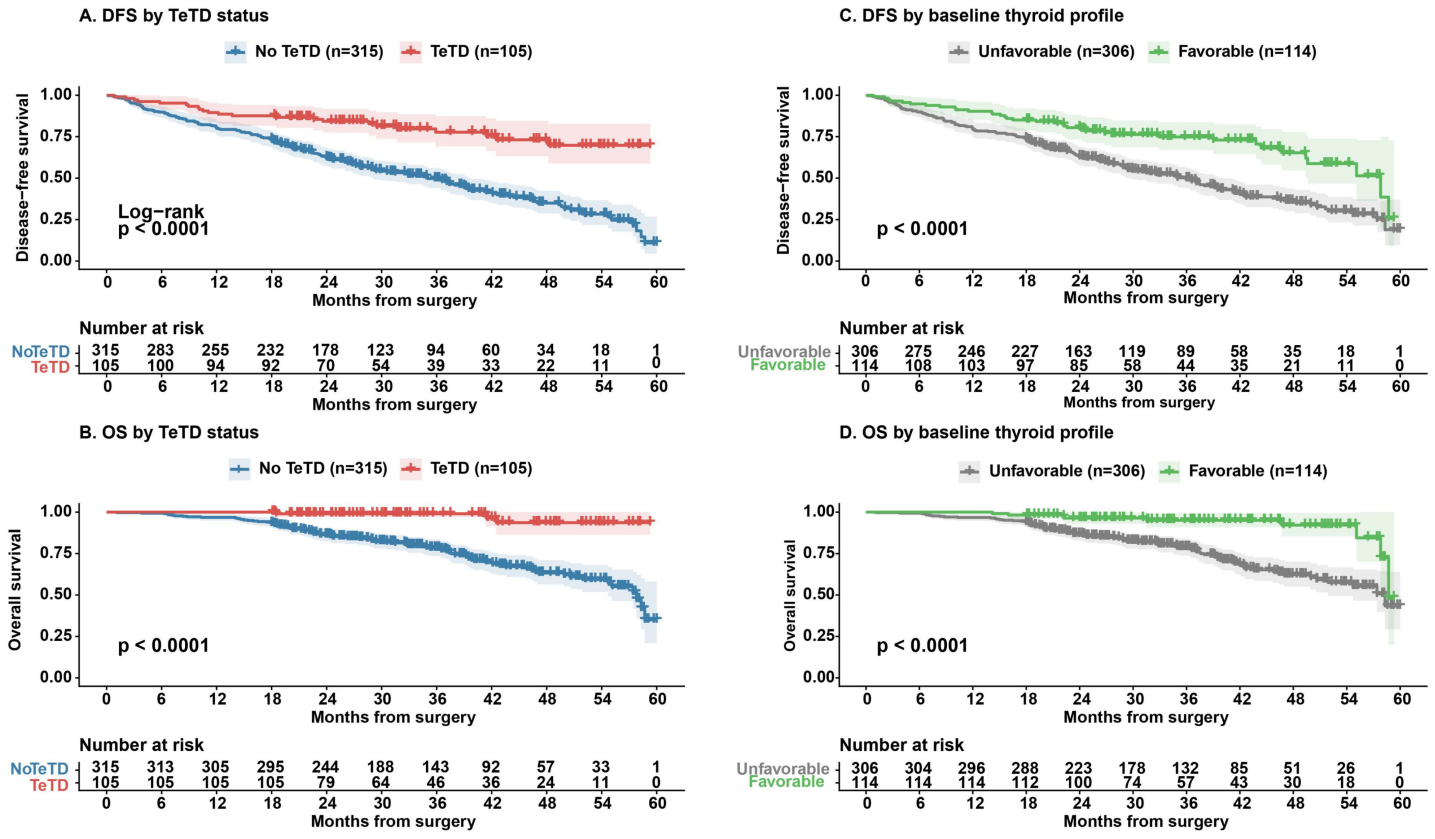
Kaplan-Meier survival curves stratified by TeTD status and by favorable thyroid profile. **(A)** Disease-free survival by TeTD status: 1-, 2-, 3-year DFS = 89.5%, 84.3%, 77.7% in TeTD versus 80.0%, 62.5%, 49.8% in non-TeTD (log-rank P<0.001). **(B)** Overall survival by TeTD status: 1-, 2-, 3-year OS = 100.0%, 99.0%, 99.0% in TeTD versus 96.8%, 86.3%, 78.4% in non-TeTD (log-rank P<0.001). **(C)** Disease-free survival by favorable baseline thyroid profile: 3-year DFS 74.8% (favorable) vs. 49.6% (unfavorable) (log-rank P<0.001). **(D)** Overall survival by favorable baseline thyroid profile: 3-year OS 95.0% vs. 78.7% (log-rank P<0.001). Numbers at risk are displayed below each panel.

The reverse Kaplan–Meier–estimated median follow-up was 39.2 months. During follow-up, 203 DFS events (48.3%) and 93 deaths (22.1%) occurred. Kaplan–Meier curves stratified by TeTD status and by favorable thyroid profile are presented in **Figure 2**. For DFS, patients with TeTD showed sustained separation from those without TeTD throughout follow-up: 1-, 2-, and 3-year DFS rates were 89.5%, 84.3%, and 77.7% in the TeTD group versus 80.0%, 62.5%, and 49.8% in the non-TeTD group (log-rank P<0.001). For OS, the corresponding 1-, 2-, and 3-year rates were 100.0%, 99.0%, and 99.0% in TeTD versus 96.8%, 86.3%, and 78.4% in non-TeTD (log-rank P<0.001). Stratification by favorable baseline thyroid profile yielded similar separation: 3-year DFS 74.8% vs. 49.6% (favorable vs. unfavorable; log-rank P<0.001) and 3-year OS 95.0% vs. 78.7% (log-rank P<0.001).

In multivariable Cox regression for DFS (**Table 6**), TeTD (adjusted HR 0.35, 95% CI 0.23–0.55; P<0.001) and favorable baseline thyroid profile (adjusted HR 0.49, 95% CI 0.34–0.71; P<0.001) remained independent favorable prognostic factors after simultaneous adjustment for clinicopathologic and tissue-biomarker variables. Other independent predictors of DFS were cT4a/b (HR 1.62; P = 0.001), cN2–3 (HR 1.35; P = 0.048), PD-L1 CPS ≥5 (HR 0.62; P = 0.002), and pCR (HR 0.29; P<0.001). In the OS multivariable model (**Table 7**), TeTD (adjusted HR0.11, 95% CI 0.03–0.35; P<0.001) and favorable thyroid profile (adjusted HR 0.24, 95% CI 0.12–0.48; P<0.001) again remained associated with significant survival benefit. cN2–3 (HR 3.11; P<0.001), age ≥65 years (HR 1.73; P = 0.015), cT4a/b (HR 1.60; P = 0.034), pCR (HR 0.27; P = 0.014), PD-L1 CPS ≥5 (HR 0.49; P = 0.003), and SII–PNI score 2 (HR 2.36; P<0.001) were the other independently significant predictors.

**Table 6:** Univariable and multivariable Cox regression for disease-free survival (DFS).

Variable	Univariable HR (95% CI)	Univariable P	Multivariable HR (95% CI)	Multivariable P
Favorable thyroid profile (Yes vs. No)	0.47 (0.32–0.67)	<0.001	0.49 (0.34–0.71)	<0.001
Treatment-emergent thyroid dysfunction (Yes vs. No)	0.31 (0.20–0.48)	<0.001	0.35 (0.23–0.55)	<0.001
Age (≥65 vs. <65)	1.16 (0.87–1.54)	0.313	1.19 (0.88–1.60)	0.265
Sex (Male vs. Female)	0.97 (0.72–1.30)	0.834	—	—
BMI (≥24 vs. <24 kg/m²)	0.93 (0.70–1.23)	0.613	—	—
cT stage (cT4a/b vs. cT2/3)	1.53 (1.16–2.04)	0.003	1.62 (1.21–2.16)	0.001
cN stage (cN2–3 vs. cN0-1)	1.61 (1.20–2.16)	0.001	1.35 (1.00–1.82)	0.048
PD-L1 (CPS ≥5 vs. <5)	0.58 (0.43–0.78)	<0.001	0.62 (0.46–0.84)	0.002
MSI-H vs. MSS	0.87 (0.51–1.51)	0.627	0.84 (0.47–1.50)	0.552
pCR (Yes vs. No)	0.22 (0.13–0.36)	<0.001	0.37 (0.22–0.63)	<0.001
MPR (Yes vs. No)	0.43 (0.31–0.59)	<0.001	—	—
SII–PNI score 2 vs. 0–1	1.61 (1.16–2.24)	0.005	1.54 (1.10–2.16)	0.012
Regimen (XELOX vs. SOX)	1.29 (0.97–1.70)	0.077	1.18 (0.89–1.56)	0.246
Differentiation (Poor/SRC vs. Well/Mod)	0.96 (0.73–1.27)	0.770	—	—

HR, hazard ratio; CI, confidence interval. Proportional hazards assumption was verified by Schoenfeld residuals (global P>0.10).

**Table 7:** Univariable and multivariable Cox regression for overall survival (OS).

Variable	Univariable HR (95% CI)	Univariable P	Multivariable HR (95% CI)	Multivariable P
Favorable thyroid profile (Yes vs. No)	0.24 (0.12–0.48)	<0.001	0.24 (0.12–0.48)	<0.001
Treatment-emergent thyroid dysfunction (Yes vs. No)	0.10 (0.03–0.30)	<0.001	0.11 (0.03–0.35)	<0.001
Age (≥65 vs. <65)	1.79 (1.19–2.70)	0.005	1.94 (1.26–3.00)	0.003
Sex (Male vs. Female)	1.68 (1.03–2.73)	0.037	—	—
BMI (≥24 vs. <24 kg/m²)	1.00 (0.66–1.52)	0.985	—	—
cT stage (cT4a/b vs. cT2/3)	1.25 (0.83–1.89)	0.289	1.51 (0.98–2.33)	0.064
cN stage (cN2–3 vs. cN0-1)	2.37 (1.48–3.80)	<0.001	2.26 (1.40–3.66)	<0.001
PD-L1 (CPS ≥5 vs. <5)	0.78 (0.51–1.19)	0.241	0.78 (0.50–1.23)	0.286
MSI-H vs. MSS	1.04 (0.48–2.24)	0.927	0.62 (0.27–1.45)	0.270
pCR (Yes vs. No)	0.14 (0.05–0.37)	<0.001	0.27 (0.10–0.77)	0.014
MPR (Yes vs. No)	0.41 (0.25–0.65)	<0.001	—	—
SII–PNI score 2 vs. 0–1	2.64 (1.70–4.09)	<0.001	2.36 (1.51–3.68)	<0.001
Regimen (XELOX vs. SOX)	1.35 (0.90–2.03)	0.153	1.15 (0.75–1.74)	0.521
Differentiation (Poor/SRC vs. Well/Mod)	0.70 (0.47–1.06)	0.090	—	—

OS analyses based on 93 deaths during follow-up. The univariable and multivariable hazard ratios for a favorable thyroid profile coincide at two-decimal precision and differ only beyond the second decimal; on audit both were confirmed correctly computed. To distinguish them, three-decimal estimates (0.240, 95% CI 0.120–0.477, univariable; 0.238, 95% CI 0.118–0.481, multivariable) are reported here. Because the number of deaths within the TeTD group is small, the OS hazard ratio for TeTD was additionally estimated with a Firth-penalized Cox model as a sensitivity analysis, which yielded a consistent estimate (HR 0.13, 95% CI 0.04–0.33) and confirms the value reported in the Abstract, text, and **Table 7**.

### Dynamic thyroid trajectories

**Figure 3:**
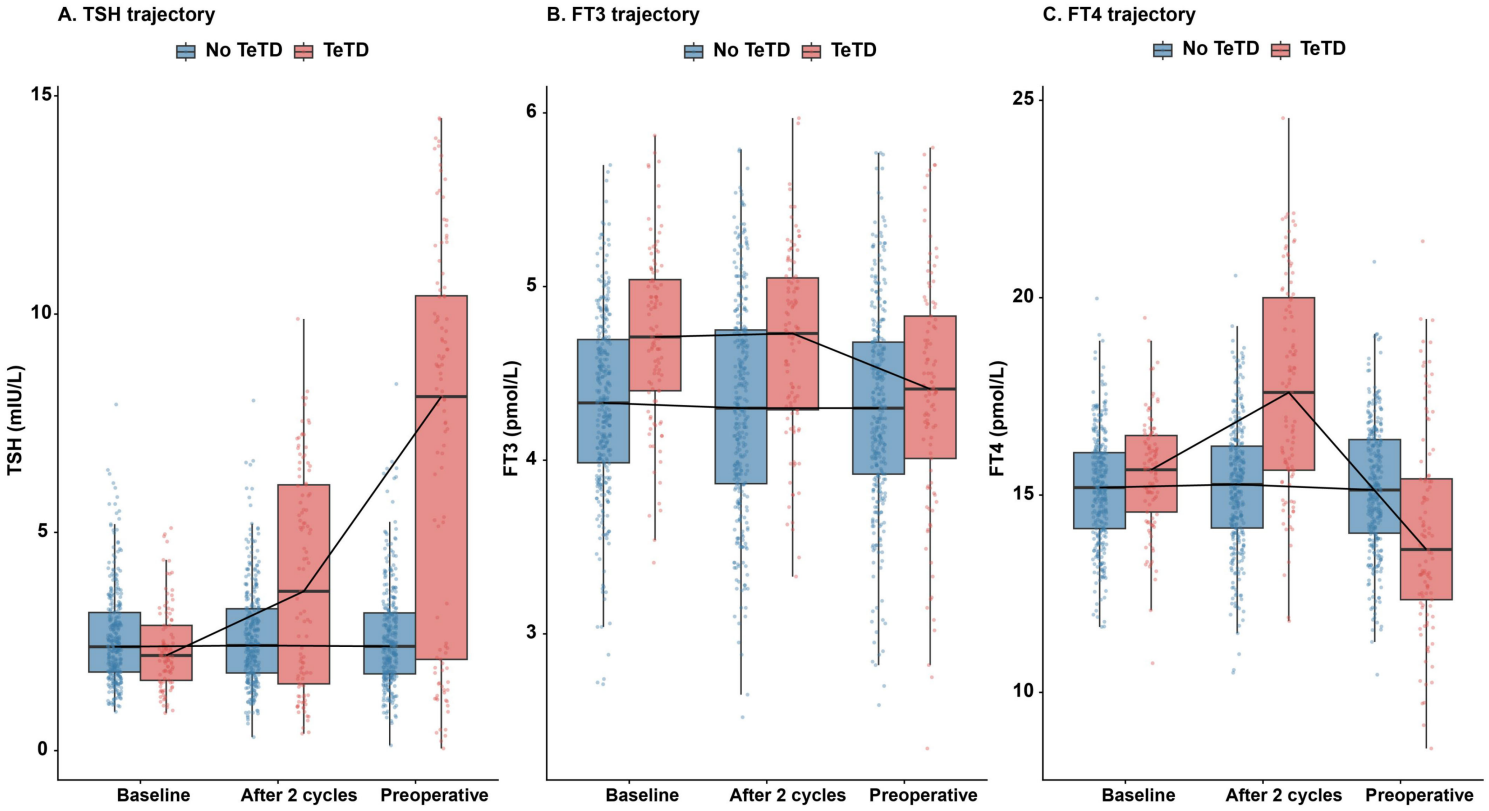
Dynamic thyroid hormone trajectories (TSH, FT3, FT4) from baseline, to after two cycles, to preoperative assessment, stratified by TeTD status. **(A)** TSH trajectory. In patients without TeTD, TSH remained stable across the three timepoints (median 2.38, 2.41, 2.39 mIU/L), whereas in patients with TeTD, TSH rose from a median of 2.18 mIU/L at baseline to 3.65 mIU/L after two cycles and 8.11 mIU/L preoperatively. **(B)** FT3 trajectory. FT3 remained stable in the No-TeTD group (median 4.33, 4.30, 4.30 pmol/L) and showed comparatively modest change in the TeTD group. **(C)** FT4 trajectory. FT4 remained stable in the No-TeTD group (median 15.19, 15.27, 15.13 pmol/L), whereas in the TeTD group FT4 declined from a median of 15.64 pmol/L at baseline to 13.62 pmol/L preoperatively, consistent with the dominant hypothyroid-direction shift. Boxes show the median and interquartile range and whiskers the distribution; subtype-specific overlays distinguish hypothyroid, thyrotoxic, and biphasic patterns. Between-group comparison at each timepoint was by Mann -Whitney U test; all P<0.001 for TSH at the preoperative timepoint.

Serial thyroid function trajectories from baseline to after two cycles to preoperative assessment are presented in **Figure 3**. In patients without TeTD, median TSH, FT3, and FT4 remained stable across all three timepoints (TSH median 2.38, 2.41, and 2.39 mIU/L; FT3 4.33, 4.30, and 4.30 pmol/L; FT4 15.19, 15.27, and 15.13 pmol/L). In contrast, patients who developed TeTD showed substantial and clinically meaningful trajectory shifts, dominated by hypothyroid-direction changes: TSH rose from a median of 2.18 mIU/L at baseline to 3.65 mIU/L after two cycles and 8.11 mIU/L preoperatively; FT4 declined from 15.64 pmol/L at baseline to 13.62 pmol/L preoperatively. These shifts were heterogeneous by TeTD subtype, with hypothyroid subtypes driving the rising-TSH/falling-FT4 pattern, and thyrotoxic and biphasic subtypes showing transient TSH suppression and FT4 elevation followed in some cases by a hypothyroid phase, consistent with destructive thyroiditis evolving into permanent thyroid hypofunction.

### Subgroup analyses

The DFS benefit associated with TeTD was directionally consistent across all prespecified subgroups (**Table 8**). Treatment-effect heterogeneity was modest. The cN-stage interaction approached but did not reach the conventional threshold (P-interaction=0.062): patients with cN0–1 disease appeared to derive a larger relative benefit (HR 0.18, 95% CI 0.08–0.42) than those with cN2–3 disease (HR 0.45, 95% CI 0.27–0.75), suggesting that TeTD carries proportionally more information when nodal burden is lower. The MSI-H subgroup (n=33) was small with a wide confidence interval, although the point estimate remained directionally favorable. No interaction with regimen was observed (P-interaction=0.160), indicating that the predictive value of TeTD is not specific to either SOX or XELOX backbone. Efficacy and TeTD incidence were additionally examined across the five individual PD-1 inhibitors (**Supplementary Table 1**). pCR and MPR were broadly comparable across agents, and the association between TeTD and pCR showed no significant interaction with the specific inhibitor used (P-interaction 0.81), indicating that the predictive signal was not driven by any single agent. The pembrolizumab and toripalimab subsets were small, so agent-specific estimates are exploratory.

**Table 8:** Subgroup analysis for DFS by TeTD status.

Subgroup	N	No TeTD (n)	TeTD (n)	HR (95% CI)	P	P-interaction
Overall	420.0	315.0	105.0	0.31 (0.20–0.48)	<0.001	—
Age	nan	nan	nan	nan	nan	0.436
<65 years	273.0	205.0	68.0	0.36 (0.21–0.61)	<0.001	nan
≥65 years	147.0	110.0	37.0	0.25 (0.11–0.54)	<0.001	nan
Sex	nan	nan	nan	nan	nan	0.876
Female	139.0	100.0	39.0	0.35 (0.17–0.71)	0.003	nan
Male	281.0	215.0	66.0	0.30 (0.17–0.52)	<0.001	nan
BMI	nan	nan	nan	nan	nan	0.797
<24 kg/m²	241.0	178.0	63.0	0.33 (0.19–0.56)	<0.001	nan
≥24 kg/m²	179.0	137.0	42.0	0.29 (0.14–0.60)	<0.001	nan
cT stage	nan	nan	nan	nan	nan	0.894
cT2/3	188.0	138.0	50.0	0.29 (0.14–0.60)	<0.001	nan
cT4a/b	232.0	177.0	55.0	0.32 (0.19–0.56)	<0.001	nan
cN stage	nan	nan	nan	nan	nan	0.062
cN0-1	177.0	123.0	54.0	0.18 (0.08–0.42)	<0.001	nan
cN2-3	243.0	192.0	51.0	0.45 (0.27–0.75)	0.002	nan
PD-L1 CPS	nan	nan	nan	nan	nan	0.831
CPS <5	245.0	191.0	54.0	0.33 (0.19–0.56)	<0.001	nan
CPS ≥5	175.0	124.0	51.0	0.30 (0.14–0.62)	0.001	nan
MSI	nan	nan	nan	nan	nan	0.931
MSS	387.0	288.0	99.0	0.31 (0.20–0.49)	<0.001	nan
MSI-H	33.0	27.0	6.0	0.43 (0.05–3.34)	0.418	nan
Favorable thyroid profile	nan	nan	nan	nan	nan	0.556
Unfavorable	306.0	229.0	77.0	0.28 (0.17–0.45)	<0.001	nan
Favorable	114.0	86.0	28.0	0.39 (0.14–1.10)	0.074	nan
Tumor location	nan	nan	nan	nan	nan	0.893
Mid/Lower	292.0	214.0	78.0	0.31 (0.18–0.51)	<0.001	nan
Upper/GEJ	128.0	101.0	27.0	0.34 (0.14–0.78)	0.011	nan
Regimen	nan	nan	nan	nan	nan	0.160
SOX+PD-1	247.0	184.0	63.0	0.25 (0.13–0.45)	<0.001	nan
XELOX+PD-1	173.0	131.0	42.0	0.47 (0.25–0.88)	0.019	nan

HRs reflect the comparison of TeTD vs. no-TeTD within each subgroup. P-interaction tested by inclusion of multiplicative interaction terms in the Cox model.

### Sensitivity analyses

To confirm the robustness of the thyroid-outcome associations, prespecified sensitivity analyses were conducted excluding patients with subclinical hypothyroidism at baseline, low-FT3 syndrome at baseline, autoantibody-positive patients, MSI-H patients, patients with grade 3–4 irAEs, and within each chemotherapy backbone (**Table 9**). After exclusion of subclinical hypothyroidism, the adjusted OR for pCR by TeTD was 3.32 (P<0.001) and by favorable profile 7.13 (P<0.001); after exclusion of autoantibody-positive patients, the adjusted OR for pCR by TeTD remained robust at 4.60 (P<0.001) and the DFS HR for TeTD was 0.49 (P = 0.041). Within each regimen subset, effects remained directionally and quantitatively consistent (SOX-only: pCR adjusted OR 3.40 for TeTD and 4.93 for favorable profile; XELOX-only: pCR adjusted OR 2.82 for TeTD and 9.59 for favorable profile). These analyses indicate that the thyroid–outcome associations are not driven by confounding from preexisting autoimmunity, by misclassification of biomarker-defined favorable subsets, by toxicity-related discontinuation, or by regimen-specific effects.

**Table 9:** Sensitivity analyses for pCR and DFS.

Sensitivity analysis	N	pCR adj OR: TeTD	pCR adj OR: favorable baseline	DFS adj HR: TeTD	DFS adj HR: favorable baseline
Full cohort	420	3.25 (1.83–5.80); P<0.001	6.20 (3.46–11.11); P<0.001	0.30 (0.20–0.47); P<0.001	0.41 (0.28–0.58); P<0.001
Excluding subclinical hypothyroidism at baseline	394	3.32 (1.81–6.10); P<0.001	7.13 (3.86–13.19); P<0.001	0.30 (0.19–0.47); P<0.001	0.40 (0.27–0.57); P<0.001
Excluding low-FT3 syndrome at baseline	340	3.48 (1.86–6.54); P<0.001	6.44 (3.43–12.13); P<0.001	0.31 (0.19–0.49); P<0.001	0.42 (0.29–0.61); P<0.001
Excluding TPOAb/TgAb-positive patients	305	4.60 (1.93–10.93); P<0.001	5.41 (2.79–10.48); P<0.001	0.49 (0.24–0.97); P = 0.041	0.36 (0.25–0.54); P<0.001
Excluding MSI-H patients	387	2.96 (1.64–5.37); P<0.001	5.63 (3.09–10.26); P<0.001	0.30 (0.19–0.47); P<0.001	0.40 (0.27–0.58); P<0.001
Excluding patients with grade 3–4 irAE	391	2.85 (1.55–5.26); P<0.001	6.11 (3.34–11.15); P<0.001	0.32 (0.20–0.50); P<0.001	0.39 (0.27–0.57); P<0.001
SOX+PD-1 subgroup	247	3.40 (1.57–7.36); P = 0.002	4.93 (2.28–10.68); P<0.001	0.22 (0.12–0.40); P<0.001	0.45 (0.28–0.73); P = 0.001
XELOX+PD-1 subgroup	173	2.82 (1.13–7.02); P = 0.026	9.59 (3.63–25.33); P<0.001	0.50 (0.26–0.95); P = 0.033	0.30 (0.17–0.55); P<0.001

Each scenario refits the multivariable logistic (pCR) and Cox (DFS) models within the indicated subset, with the same set of clinical covariates. Estimates remain directionally consistent across all scenarios, supporting the robustness of the thyroid-outcome associations.

### Construction and validation of a thyroid-inclusive nomogram for pCR

**Figure 4:**
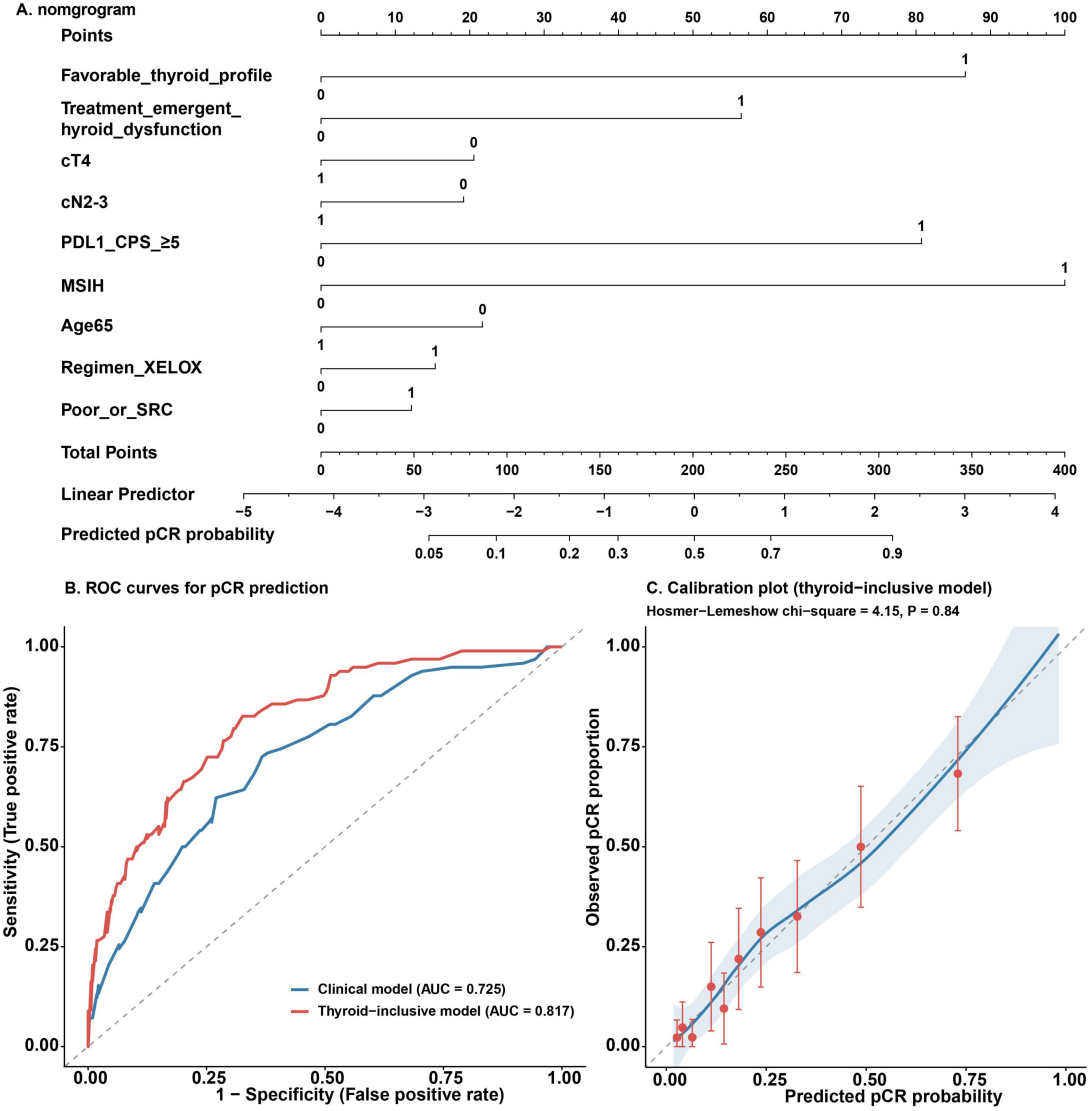
Performance of the thyroid-inclusive nomogram for pCR prediction. **(A)** Nomogram incorporating favorable baseline thyroid profile, TeTD, cT stage, cN stage, PD-L1 CPS, MSI status, regimen, age, and differentiation. Each predictor maps to a points axis; total points are summed and read off the predicted pCR probability axis. **(B)** Receiver-operating characteristic (ROC) curves comparing the thyroid-inclusive model (AUC 0.82, 95% CI 0.77-0.86) with the clinical staging-only model (AUC 0.73, 95% CI 0.67-0.78); bootstrap-based DeLong-equivalent P<0.001. **(C)** Calibration plot of the thyroid-inclusive model: predicted versus observed pCR probability across deciles of predicted risk; Hosmer-Lemeshow chi-square = 4.15, P = 0.84. The diagonal reference line indicates perfect calibration.

Based on the multivariable logistic model, a nomogram for individualized pCR prediction was developed incorporating favorable baseline thyroid profile, TeTD, cT stage, cN stage, PD-L1 CPS, MSI status, age, regimen, and differentiation (**Figure 4A**). The thyroid-inclusive nomogram achieved an AUC of 0.82 (95% CI 0.77–0.86), significantly higher than a clinical staging-only baseline model (cT, cN, PD-L1, MSI, age, regimen) which yielded an AUC of 0.73 (95% CI 0.67–0.78) (bootstrap DeLong-equivalent P<0.001; **Figure 4B**). Calibration plots demonstrated good agreement between predicted and observed pCR probabilities across the full risk spectrum, supported by a non-significant Hosmer–Lemeshow goodness-of-fit test (χ²=4.15, P = 0.84; **Figure 4C**). The thyroid-inclusive model therefore both improved discrimination and retained adequate calibration, supporting its clinical interpretability.

## Discussion

In this multicenter retrospective cohort of 420 patients with locally advanced gastric and GEJ adenocarcinoma treated with neoadjuvant PD-1 inhibitor plus SOX or XELOX chemotherapy, two simple thyroid-axis variables — a prespecified favorable baseline thyroid profile and treatment-emergent thyroid dysfunction — provided clinically meaningful, independent, and complementary prediction of pathological response and long-term survival. Patients with a favorable baseline thyroid profile were nearly three times as likely to achieve pCR (43.0% vs. 16.0%; adjusted OR 6.08), and patients who developed TeTD during neoadjuvant therapy were twice as likely to achieve pCR (38.1% vs. 18.4%; adjusted OR 3.26) as their respective comparators. With a median follow-up of 39.2 months, the 3-year DFS rate was 77.7% in TeTD versus 49.8% in non-TeTD patients (adjusted HR 0.35, P<0.001), and 3-year OS was 99.0% versus 78.4% (adjusted HR 0.26, P<0.001). A thyroid-inclusive nomogram outperformed conventional staging-based prediction for pCR (AUC 0.82 vs. 0.73, P<0.001) with adequate calibration. These associations were robust across all prespecified subgroups, sensitivity analyses excluding patients with preexisting subclinical hypothyroidism, autoantibody-positive patients, MSI-H patients, and patients with grade 3–4 irAEs, and were consistent within each chemotherapy backbone.

To our knowledge, this represents the largest multicenter study to date evaluating both baseline and on-treatment thyroid-axis variables jointly as biomarkers for neoadjuvant immunochemotherapy response in LAGC and GEJC treated with the two most widely used Asian regimens (SOX and XELOX). The findings extend prior observations from advanced-stage palliative ICI cohorts in non-small-cell lung, hepatocellular, and urothelial carcinomas to the early-stage perioperative setting where pathological endpoints can be directly assessed and treatment decisions are most consequential. Our cohort’s pCR rate of 23.3% and MPR rate of 42.6% are consistent with the pCR rates reported in DANTE, MATTERHORN, KEYNOTE-585, and Chinese RESOLVE-platform trials, providing a credible clinical context within which to interpret the present thyroid findings (5, 6). Our 3-year DFS of approximately 56% in the overall cohort and 78% in TeTD patients aligns with contemporary international standards for high-quality neoadjuvant immunochemotherapy delivery.

The biological rationale for an association between thyroid-axis status and ICI response is multifaceted. First, a favorable baseline thyroid profile likely reflects preserved endocrine-metabolic reserve, adequate systemic immune competence, and absence of cancer-related cachexia or systemic inflammation that would otherwise impair T-cell function and antitumor immunity. Low-FT3 syndrome, in particular, is a recognized signature of catabolism, malnutrition, and chronic inflammation in advanced cancer, and is associated with poor outcomes across tumor types. (33, 34) Second, treatment-emergent thyroid dysfunction has been increasingly interpreted as a clinical biomarker of pharmacodynamic immune activation by PD-1 blockade (31, 33, 35–40). The thyroid is uniquely susceptible to immune attack during ICI therapy because it is rich in MHC class I expression, possesses an extensive blood supply that delivers reinvigorated cytotoxic T cells, and contains autoreactive T-cell repertoires that are normally held in check by peripheral tolerance mechanisms that PD-1 blockade releases. The frequent involvement of preexisting TPOAb/TgAb in our TeTD cohort (42.9% and 28.6%, respectively), compared with the very low prevalence in non-TeTD patients (6.0% and 10.2%), suggests that latent thyroid autoimmunity primes the response, and is uncovered by checkpoint blockade. The same loss of peripheral tolerance that drives TeTD may simultaneously license effective antitumor immunity against neoantigens, explaining the observed coupling between thyroid dysfunction and pathological response.

An important practical implication of our findings is that, unlike many biomarkers associated with severe ICI toxicity, TeTD does not appear to come at the cost of substantial non-thyroid morbidity. Skin, hepatic, pulmonary, gastrointestinal, and renal irAEs were not significantly more frequent in TeTD than in non-TeTD patients, and treatment-discontinuation rates due to irAE were not significantly increased. The grade 3–4 irAE rate was higher in TeTD primarily because overt hypothyroidism itself meets grade 3 criteria, but levothyroxine replacement is straightforward, well tolerated, and does not require ICI interruption in most cases. This benign-toxicity profile, alongside the strong and consistent efficacy signal, distinguishes TeTD from biomarkers associated with hyper-progressive immune activation, and supports its practical utility for patient counseling and treatment-intensification decisions.

Our observation that favorable baseline thyroid profile and TeTD remained independently significant after mutual adjustment, and after adjustment for PD-L1 and MSI status, is mechanistically informative. A favorable profile likely captures pretreatment host fitness, while TeTD captures the on-treatment pharmacodynamic activation of immune tolerance breakdown. These two signals appear to act through partially overlapping but non-redundant pathways, consistent with the immunological framework outlined above. Their additive predictive value is reflected in the substantial AUC improvement of the thyroid-inclusive nomogram (0.82) over the staging-only model (0.73). For comparison, published nomograms for neoadjuvant ICI response in gastric cancer have typically reported AUCs in the 0.70–0.78 range when relying solely on conventional staging and tissue biomarkers, suggesting that thyroid-axis variables can meaningfully complement existing risk-stratification frameworks at trivial incremental cost (16, 17).

Several limitations of our study warrant consideration. First, this was a retrospective cohort, with all the inherent risks of unmeasured confounding. Although we adjusted for the most clinically relevant covariates, including tumor stage, PD-L1 CPS, MSI status, and SII–PNI score, residual confounding remains possible. Second, the temporal sequence between TeTD and pCR raises the potential for immortal-time bias: patients who develop TeTD must have survived long enough to receive sufficient ICI exposure for the irAE to manifest. However, because all patients were required to receive a full four cycles of neoadjuvant therapy and proceed to surgery, with the median TeTD onset at cycle 2 (well before surgery), the magnitude of this bias is expected to be limited. Landmark analyses re-anchored at 3 and 6 months after surgery yielded disease-free survival hazard ratios for TeTD of 0.31 (95% CI 0.19–0.48) and 0.29 (95% CI 0.18–0.47), and the proportional-hazards assumption for TeTD was satisfied (global Schoenfeld residual P 0.15). These results, detailed in **Supplementary Table 2**, were directionally and quantitatively consistent with the primary model, supporting the reliability of TeTD as a biomarker despite its on-treatment ascertainment. Third, although the cohort spanned five tertiary centers within Hebei Province, all patients were Chinese, and external validation in other ethnic populations and in non-Asian regimen backbones (e.g., FLOT) is needed before generalization. Fourth, our analyses did not include genomic correlates such as TMB, neoantigen burden, or HLA typing, all of which may interact with thyroid-axis status. Fifth, although pathological response was assessed by independent gastrointestinal pathologists at each center, central pathology re-review was not performed. Sixth, thyroid hormones were measured on center-specific FT3 and FT4 chemiluminescence immunoassays harmonized through the regional accreditation network rather than by a single reference method, and some laboratories elsewhere report total T3 in preference to FT3; residual inter-assay variability and the FT3-versus-total-T3 distinction may limit direct transportability of the numeric cutoffs to other settings, although the prespecified band was applied uniformly within this cohort.

These findings support several clinical and research directions. Clinically, given that thyroid function tests are inexpensive, ubiquitously available, and non-invasive, our results support including baseline thyroid panel testing in the standard pretreatment evaluation of LAGC/GEJC patients being considered for neoadjuvant immunochemotherapy. Patients with a favorable baseline profile and those who develop early TeTD may be appropriate candidates for treatment intensification or organ-preservation strategies, while those without either signal may benefit from alternative or combinatorial approaches. From a research perspective, prospective validation in independent cohorts, integration with tumor-derived biomarkers (PD-L1, MSI, TMB) and multimodal AI-assisted prediction frameworks, and mechanistic studies dissecting the shared T-cell-receptor repertoire between thyroid and tumor antigens are warranted (16, 17, 41). In parallel, non-invasive transcriptomic liquid-biopsy panels are emerging as a means of molecular recurrence surveillance and could be combined with host-factor indices such as thyroid-axis status to refine post-treatment monitoring (42). Separately, deep-learning digital-pathology models that quantify histological features from routine sections provide an orthogonal route to recurrence-risk stratification and warrant integration with the systemic biomarkers described here (43, 44). Finally, randomized comparisons of neoadjuvant chemotherapy backbones will help define the optimal partner for immunotherapy in this setting (45).

## Conclusions

In this multicenter retrospective cohort of 420 patients with locally advanced gastric or gastroesophageal junction adenocarcinoma receiving four cycles of neoadjuvant PD-1 inhibitor plus SOX or XELOX chemotherapy, a favorable baseline thyroid profile and treatment-emergent thyroid dysfunction were independent and complementary predictors of improved pathological response and long-term survival. A thyroid-inclusive nomogram outperformed a conventional staging-only model for pCR prediction (AUC 0.82 vs. 0.73, P<0.001). These inexpensive, readily available host-factor biomarkers warrant prospective validation and consideration for incorporation into pretreatment risk stratification and individualized treatment planning for LAGC/GEJC.

## Data Availability

The raw data supporting the conclusions of this article will be made available by the authors, without undue reservation.
