# Supplementary material for: Baseline thyroid function and treatment-emergent thyroid dysfunction predict pathological response and survival after neoadjuvant PD-1 inhibitor plus platinum-based chemotherapy in locally advanced gastric and gastroesophageal junction adenocarcinoma: a multicenter cohort study
Source: Front Endocrinol (Lausanne). 2026 Jul 27;17:1872057. doi: 10.3389/fendo.2026.1872057 (PMC13453737; doi:10.3389/fendo.2026.1872057)
Supplement: Supplementary file 1 [file Table1.docx]

**Supplementary Material**

**Supplementary Table S1.** Pathological efficacy and treatment-emergent thyroid dysfunction by individual PD-1 inhibitor.

| **PD-1 inhibitor** | **No. of patients** | **pCR, n (%)** | **MPR, n (%)** | **TeTD, n (%)** |
| --- | --- | --- | --- | --- |
| Camrelizumab | 109 | 29 (26.6) | 52 (47.7) | 31 (28.4) |
| Pembrolizumab | 19 | 5 (26.3) | 11 (57.9) | 4 (21.1) |
| Sintilimab | 151 | 31 (20.5) | 60 (39.7) | 39 (25.8) |
| Tislelizumab | 94 | 23 (24.5) | 40 (42.6) | 24 (25.5) |
| Toripalimab | 47 | 10 (21.3) | 16 (34.0) | 7 (14.9) |
| **Overall** | **420** | **98 (23.3)** | **179 (42.6)** | **105 (25.0)** |

*pCR, pathological complete response; MPR, major pathological response; TeTD, treatment-emergent thyroid dysfunction.* pCR and MPR were broadly comparable across the five agents. A formal test for interaction between TeTD and the specific PD-1 inhibitor on pCR was not significant (P for interaction = 0.81), indicating that the predictive association of TeTD with pathological response was not driven by any single agent. The pembrolizumab (n = 19) and toripalimab (n = 47) subsets are small, so agent-specific estimates are exploratory.

**Supplementary Table S2.** Landmark sensitivity analyses for treatment-emergent thyroid dysfunction (TeTD) as a test for immortal-time bias.

| **Analysis** | **No. at risk** | **Events** | **HR (95% CI)** | **P** |
| --- | --- | --- | --- | --- |
| **Disease-free survival** |  |  |  |  |
| Primary (origin at surgery) | 420 | 203 | 0.31 (0.20–0.48) | <0.001 |
| Landmark at 3 months | 403 | 186 | 0.31 (0.19–0.48) | <0.001 |
| Landmark at 6 months | 383 | 166 | 0.29 (0.18–0.47) | <0.001 |
| **Overall survival** |  |  |  |  |
| Primary (origin at surgery) | 420 | 93 | 0.10 (0.03–0.30) | <0.001 |
| Landmark at 3 months | 419 | 92 | 0.10 (0.03–0.30) | <0.001 |
| Landmark at 6 months | 418 | 91 | 0.10 (0.03–0.31) | <0.001 |

*HR, hazard ratio; CI, confidence interval; TeTD, treatment-emergent thyroid dysfunction.* Each hazard ratio is from a Cox model for TeTD (yes vs. no). Landmark analyses re-anchored the survival origin at 3 and 6 months after surgery, retaining only patients event-free to the landmark. Because TeTD was fully ascertained before surgery (median onset at cycle 2 of 4), its status is effectively fixed at the survival origin. Landmark estimates were directionally and quantitatively consistent with the primary analysis. The proportional-hazards assumption for TeTD, assessed by scaled Schoenfeld residuals, was satisfied (disease-free survival, P = 0.15; overall survival, P = 0.55).
